# Response to Lu Li and colleagues

**DOI:** 10.1093/ehjcvp/pvag062

**Published:** 2026-09-01

**Authors:** Angelo Avogaro, Mauro Gori, Giuseppe Grandaliano, Massimo Iacoviello, Roberto Trevisan

**Affiliations:** Department of Medicine, University of Padova, Via Giustiniani 2, Padova 35128, Italy; Division of Cardiology, Cardiovascular Department, Papa Giovanni XXIII Hospital, Bergamo 24127, Italy; Department of Translational Medicine and Surgery, Università Cattolica del Sacro Cuore, Rome 00168, Italy; Nephrology, Dialysis and Transplantation Unit, Fondazione Policlinico Universitario A. Gemelli IRCCS, Rome 00168, Italy; Department of Medical and Surgical Sciences, University of Foggia, Foggia 71122, Italy; Endocrinology and Diabetes Unit, Azienda Ospedaliera Papa Giovanni XXIII, Bergamo 24127, Italy; Department of Medicine and Surgery, University of Milano Bicocca, Milan 20126, Italy

This commentary refers to ‘From ‘bridging specialties’ to ‘owning the patient’: rethinking accountability in Cardiovascular-Kidney-Metabolic syndrome care’ by L. Li *et al*., https://doi.org/10.1093/ehjcvp/pvag057.

In their correspondence, Lu Li and colleagues raise important points in response to our article on the appropriate implementation of sodium-glucose cotransporter-2 inhibitors (SGLT2i) in cardiovascular-kidney-metabolic (CKM) syndrome.^1^ Their points are twofold: first, who bears ultimate accountability for the holistic management of patients with CKM syndrome; and second, no single specialty explicitly mandates or incentivizes the orchestration of continuity of care. In their view, these two issues would, first, beget therapeutic inertia, as each specialist would expect another to initiate SGLT2i treatment, and second, undermine cross-specialty coordination because of the lack of adequate incentives for this holistic approach to treatment.

We agree that these are crucial considerations when managing patients who need SGLT2i. In our article, we emphasize that SGLT2i is a disease-modifying therapy, and for this reason we believe that the medical approach to patients who need it should be proactive rather than reactive, risk-based rather than organ-based, and multidisciplinary rather than specialty-specific. Although guidelines from different specialties are now remarkably aligned on this point, clinical implementation of this treatment remains far from optimal.^2^

Lu Li and colleagues propose three measures: the creation of a ‘CKM-attending physician’ with dedicated specialization; the incorporation of SGLT2i use into a cross-specialty quality-evaluation framework; and enabling trained generalists and specialists across disciplines to initiate SGLT2i therapy. Their proposals merit the following considerations.

First, it is time to base the training of physicians on the concept of systems medicine rather than single-organ medicine. In the context of CKM syndrome, systems medicine would allow a holistic perspective of the patient, appropriate integration of patient data, better personalization of treatment, and a deeper understanding of its complex pathophysiology. This proposal appears feasible.

Second, and more difficult, is the incorporation of SGLT2i into a cross-specialty quality-evaluation framework, since this approach depends heavily on each country’s national health system (NHS). The authors’ proposal would be ideal, but we see it as challenging to allocate incentives to health organizations dealing with primary prevention. In Italy, for example, only 5% of the roughly €140 billion allocated annually to the NHS goes to primary-prevention initiatives. There is little hope of changing this trend, since, at least in Italy, the economic benefits generated by preventive healthcare are frequently constrained by ‘silo budgeting’: savings achieved through reduced disease incidence remain within the Prevention Department’s budget rather than being reallocated across the healthcare system to support and expand preventive health initiatives.^3^ As a result, the NHS will continue to bear the costs of late-stage complications in patients undertreated during the early stages of CKM syndrome.

## Data Availability

None.
